# Mapping Analysis to Predict the Associated EQ-5D-3L Utility Values from the Oxford Hip Score

**DOI:** 10.3390/jcm15218268

**Published:** 2026-10-23

**Authors:** Thomas R. Williamson, Yeon Joo Lee, David H. Sochart, Gavin J. Macpherson, Paul Gaston, Andrew D. Duckworth, Chloe E. H. Scott, Nicholas D. Clement

**Affiliations:** 1 Edinburgh Orthopaedics, Royal Infirmary of Edinburgh, Edinburgh EH16 4SA, UK; 2 Usher Institute, School of Population Health Sciences, University of Edinburgh, Edinburgh EH16 4UX, UK; 3 South West London Elective Orthopaedic Centre, Epsom and St. Helier University Hospitals NHS Trust, Epsom KT18 7EG, UK; 4 Department of Mechanical Engineering, Imperial College, London SW7 2PG, UK

**Keywords:** EQ-5D, Oxford hip score, arthroplasty, outcome, predictors

## Abstract

**Purpose:** The aim was to assess mapping models to predict the EQ-5D-3L utility from the Oxford hip score (OHS) and validate these before and after total hip arthroplasty (THA). **Methods**: Separate retrospective cohorts of patients who had undergone THA in different centres were used to create (n = 3981) and validate (n = 7168) the prediction models. Patient demographics, body mass index (BMI), OHS and EQ-5D were collected preoperatively and one year postoperatively. Predictive models were formulated using generalised linear regression. **Results:** In the modelling cohort, mean OHS improved from 20.8 preoperatively to 39.6 postoperatively; the mean EQ-5D improved from 0.391 preoperatively to 0.788 postoperatively. Significant (*p* < 0.001) correlations were observed between OHS and EQ-5D preoperatively (r = 0.690) and postoperatively (r = 0.777) and for change (r = 0.611) and combined scores (r = 0.812). Four models predicting EQ-5D from OHS explained 38.5% to 65.9% of the variance. Age and BMI did not improve model fit. Predicted mean EQ-5D utilities differed statistically from observed values for most models, but all differences were below the minimal clinical important difference (MCID). Including individual OHS items marginally improved performance (<2%). External validation showed good agreement at the cohort level but lower accuracy for individual-level prediction (27.7% for change to 65.0% for postoperative scores to be within the MCID). **Conclusions:** The EQ-5D-3L utility index, on a cohort level, may be reliably estimated from the OHS. Predicted individual patient utility error beyond the MCID varied from one-third to more than two-thirds depending on the data assessed and so should not be employed for this purpose on a patient level.

## 1. Introduction

The Oxford Hip Score (OHS) is a validated, joint-specific patient-reported outcome measure (PROM) that is widely used to assess outcomes following total hip arthroplasty (THA) [1]. An alternative approach to outcome assessment is the evaluation of health-related quality of life (HRQoL) using a generic instrument such as the EQ-5D questionnaire [2]. Generic measures facilitate comparison of the impact of different interventions on HRQoL across medical specialities [3]. Each defined health state can be assigned a utility index, allowing calculation of quality-adjusted life years (QALYs) gained or lost. Cost per QALY can then be used to assess cost-effectiveness across interventions, with THA consistently shown to be among the most cost-effective procedures [4]. However, EQ-5D data are not always routinely collected, limiting the feasibility of health economic analyses.

Mapping (or cross-walking) techniques can be used to estimate EQ-5D utilities from alternative outcome measures, such as the OHS [5,6]. While such approaches inevitably introduce uncertainty and information loss and are not a substitute for direct measurement, they provide a pragmatic solution when EQ-5D data are unavailable [7]. To date, two studies have mapped EQ-5D utilities from the OHS [8,9]. One was conducted in Spain and used a country-specific value set, limiting applicability to the UK population [9]. The United Kingdom (UK)-based study by Pinedo-Villanueva et al. mapped EQ-5D-3L from the OHS and demonstrated limited predictive accuracy (mean absolute error 0.20 to 0.23), with 58% of predictions showing clinically meaningful differences on external validation [8]. Their model also combined preoperative and postoperative data, despite differing score distributions. To the authors’ knowledge, no previous study has mapped the change in EQ-5D utility from preoperative to postoperative OHS, which is essential for estimating QALY gains in cost-utility analyses. Existing crosswalk techniques based on item-level prediction or response mapping can result in complex models, limiting their utility; thus, parsimonious, simpler models may be preferred if predictive ability is not sacrificed.

This study aimed to create models to predict the EQ-5D three-level utility from the OHS both pre- and postoperatively, as well as to determine whether the individual OHS item responses improved the models, and to externally validate these in patients undergoing THA.

## 2. Methods

This study was classed as a service evaluation and did not meet the criteria requiring ethical approval. The project was registered with both institutions’ audit departments and was conducted in accordance with the Declaration of Helsinki and the guidelines for Good Clinical Practice. The ‘Mapping onto Preference-based measures reporting Standards’ (MAPS) statement was followed; the MAPS checklist is presented in Supplement Table S1 [10]. The study methodology comprised four steps: firstly, the association between the EQ-5D and OHS was assessed; secondly, the models to predict the EQ-5D index from the OHS score were created using a single dataset; third, how EQ-5D prediction may be improved through the addition of demographic characteristics and individual OHS items to the models was assessed; and fourth, the finalised models were validated using an external dataset, comparing predicted EQ-5D with observed EQ-5D on both a patient and cohort level.

Patients were identified retrospectively from two established arthroplasty databases held at the Southwest London Elective Orthopaedic Centre (SWLEOC) and the Royal Infirmary of Edinburgh (RIE). The data from the RIE were used to construct the prediction models (Modelling Cohort), and the data from SWLEOC were used to validate the models (Validation Cohort), which was previously described [11]. At the RIE, 6603 patients were eligible for inclusion; 3852 patients (58.3%) undergoing a THA completed preoperative PROMS (both OHS and EQ-5D) and 3981 (60.3%) completed postoperative PROMS during a 10-year period (January 2013 to December 2022). For six years (January 2016 to December 2021), 7168 patients undergoing primary THA at SWLEOC completed preoperative PROMs and 6101 completed postoperative PROMs. Where patients underwent bilateral THA, only the first procedure was included, to maintain statistical independence [12].

Patient demographics and body mass index (BMI) were recorded preoperatively, and preoperative and 1-year postoperative PROMs were collected at both centres. The EQ general health questionnaire evaluates five dimensions, which include mobility, self-care, usual activities, pain/discomfort and anxiety/depression. The 3L version of the EuroQoL questionnaire was used, with the responses to the five domains being recorded at three levels of severity [2]. An individual patient’s health state can be reported based on a three-digit code for each domain, resulting in 243 possible health states [13]. Each health state was converted to a single summary index by applying a weighting. These are specific to the UK population and are based on a time trade-off technique. This index is on a scale of −0.584 to 1, where one represents perfect health, zero represents being dead, and negative values represent a state perceived as worse than death [14]. A minimal clinically important difference (MCID) is the smallest change of a score to be of importance and has been defined at 0.101 for the EQ-5D following THA [15]. The OHS consists of twelve questions that were assessed using a Likert scale with values from 0 to 4; a summative score is then calculated where 48 is the best possible score (least symptomatic), and 0 is the worst possible score (most symptomatic) [1,16].

### Statistical Analysis

Statistical analysis was performed using the Statistical Package for Social Sciences version 17.0 (SPSS Inc., Chicago, IL, USA). Simple descriptive analyses were undertaken. Student’s *t*-tests (paired and unpaired) were used to compare linear variables between groups. Dichotomous variables were assessed using a Chi-Square test. Complete-case analysis was conducted for modelling change in scores (as paired pre- and postoperative PROMs were required to determine change), whilst pre- and postoperative models were defined from cases where complete preoperative or complete postoperative PROMs were available, respectively. Correlations between OHS and EQ-5D preoperatively, postoperatively and for change scores were assessed using Pearson’s tests, and corresponding r values were presented.

In the modelling cohort, linear regression (Ordinary Least Squares) analyses were used to map the EQ-5D utility to the OHS pre- and postoperatively and for change in scores. Regression analyses were repeated, including adjustments for patient demographics, and improvement in model performance was assessed. The twelve individual OHS items were also added to the predictive models, and change in model performance was compared with the total score-only models. The final models were then used to predict the EQ-5D utility for both the modelling cohort (RIE) and the validation cohort (SWLEOC) and compared with observed values. Comparisons in fit and performance between the modelling and validation cohorts were made. A *p*-value of <0.05 was defined as statistically significant.

## 3. Results

**Figure 1:**
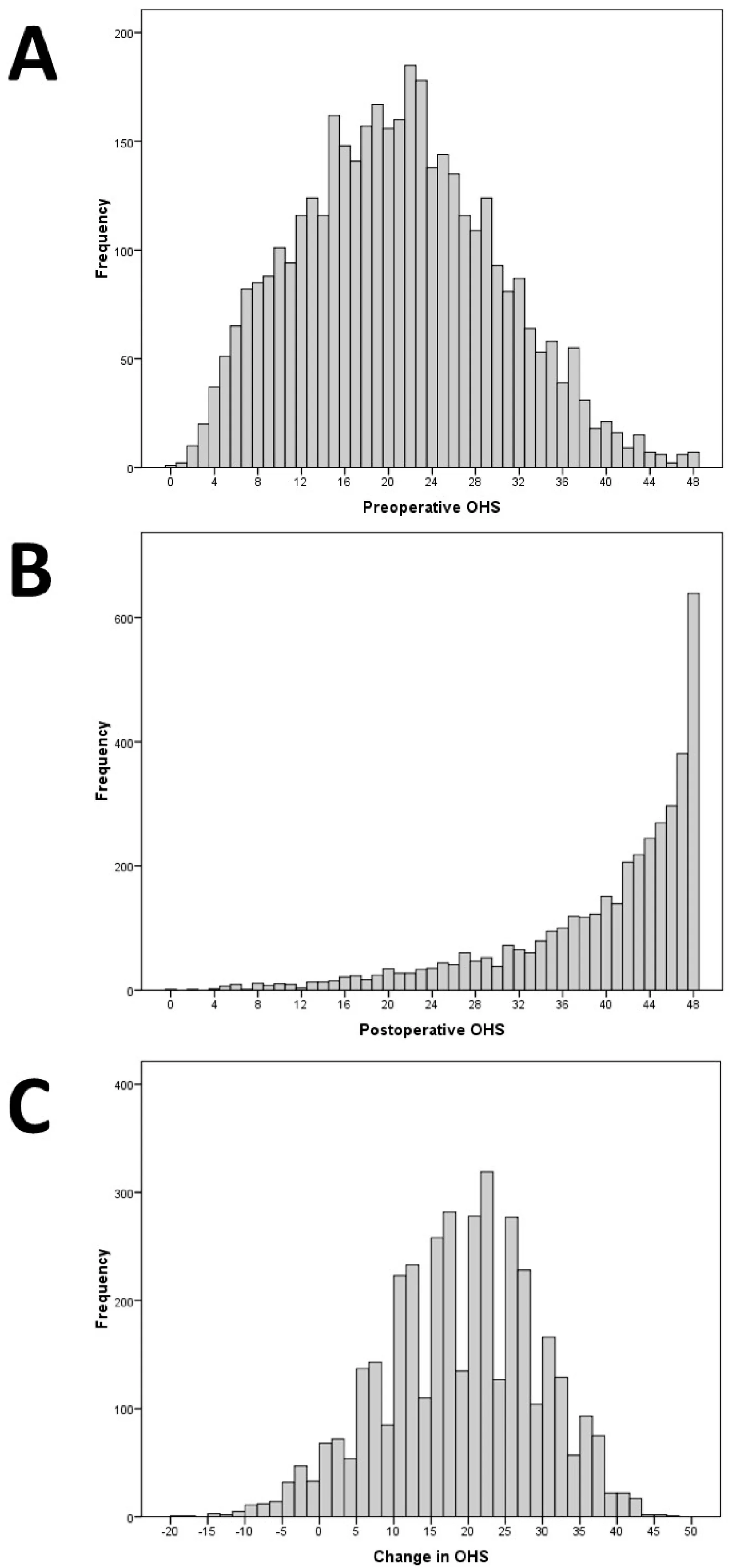
Histogram illustrating the distribution of the pre- (**A**) and postoperative (**B**) and change in (**C**) Oxford hip score (OHS) after total hip arthroplasty.

**Figure 2:**
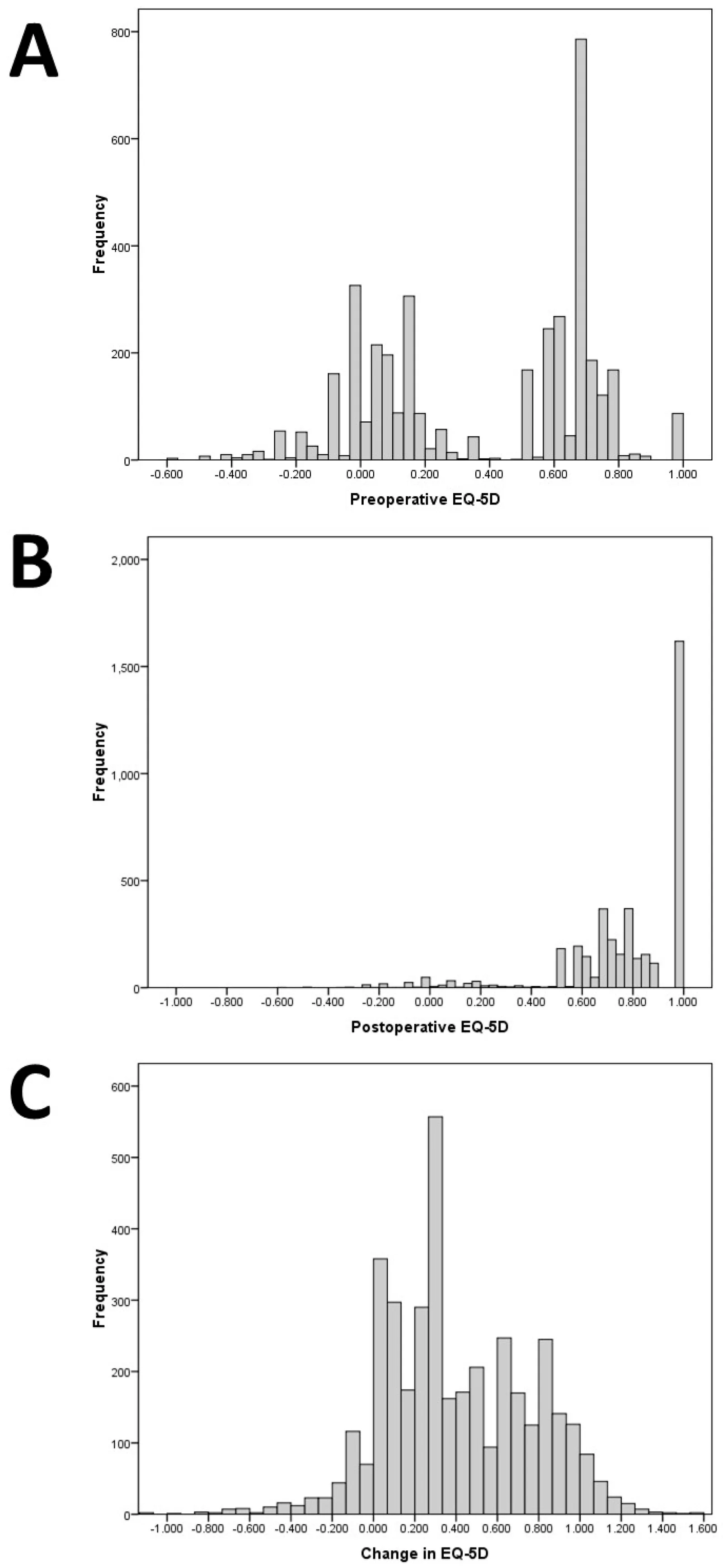
Histogram illustrating the distribution of the pre- (**A**) and postoperative (**B**) and change in (**C**) EQ-5D after total hip arthroplasty.

The mean preoperative OHS for the modelling cohort was 20.8 (SD 9.0), which improved to a mean of 39.6 (SD 9.1) at one year postoperatively, with a mean change of 18.8 (SD 10.4) points. The preoperative OHS and change in OHS data were normally distributed, but the postoperative score was left-skewed, with a ceiling effect (Figure 1). The mean preoperative EQ-5D was 0.391 (SD 0.341), which improved to a mean of 0.788 (SD 0.362) at one year postoperatively, with a mean change of 0.397 (SD 0.362) points. A bimodal distribution was observed in the preoperative EQ-5D index, a normal distribution for the change, and a left-skewed distribution in the postoperative EQ-5D, demonstrating a ceiling effect (Figure 2).

There was a moderate-to-strong correlation between the OHS and EQ-5D index preoperatively (r = 0.690, *p* < 0.001) and postoperatively (r = 0.777, *p* < 0.001), as well as for the change (r = 0.611, *p* < 0.001) in the scores. For the combined preoperative and postoperative scores, the correlation was 0.812 (*p* < 0.001).

### 3.1. Modelling: Establishing the Models

Due to differing data distributions, four models were developed to predict EQ-5D utility from the OHS: A model for preoperative score prediction, a model for postoperative score prediction, a model for change in score prediction, and a combined model incorporating both preoperative and postoperative scores. Generalised linear regression demonstrated a significant association between OHS and EQ-5D, explaining between 38.5% and 65.9% of the variance across models (Table 1).

### 3.2. Modelling: The Impact of Individual OHS Questions and Demographic Characteristics

Including individual OHS item responses in the models estimating EQ-5D utility for preoperative, postoperative, and change scores improved model performance (Table 2). The proportion of EQ-5D variance explained by the models (R^2^) increased by 1.0% to 1.9%, relative to the models using the total OHS alone (Table 1). Items relating to limping, night pain, and putting on socks were not significantly associated with preoperative EQ-5D or change in EQ-5D, whereas the question relating to transport was not associated postoperatively. In contrast, question one (pain) and question five (ability to shop) consistently ranked among the strongest predictors (highest beta coefficients) across preoperative, postoperative, and change models, contributing most to improved predictive performance (Table 2).

When age, sex, and BMI were included as covariates, only age (β = 0.001, 95% CI 0.000 to 0.001, *p* = 0.018) and BMI (β = 0.002, 95% CI 0.000 to 0.004, *p* = 0.040) showed statistically significant associations with postoperative and change in EQ-5D, respectively. However, inclusion of these variables did not meaningfully improve model fit (R^2^) and were therefore excluded.

### 3.3. Modelling: Finalised Predictive Models

Neither OHS individual items nor patient demographic characteristics were included in the final models, as they offered minimal improvements in model fit (Delta R^2^ 0.01 to 0.019) and increased complexity. The four final models were used to derive equations predicting EQ-5D index values, as below:

EQ-5D_preoperative_ = (OHS_preoperative_ × 0.026) − 0.142

EQ-5D_postoperative_ = (OHS_postoperative_ × 0.022) − 0.075

EQ-5D_change_ = OHS_change_ × 0.022

EQ-5D_combined_ = (OHS_combined_ × 0.022) − 0.087

Using these equations, the predicted ED-5D was calculated from the OHS at the differing timepoints and for the change in the score (Table 3). Compared with the observed score, there was a significantly greater predicted preoperative, postoperative, and change in the EQ-5D index, whereas this was less with the combined data (Table 4). However, the mean differences between 0.007 and 0.015 were all less than the MCID.

### 3.4. Validation of Prediction Models

The patient demographics and OHS and EQ-5D scores were similar between the modelled cohort and validation cohort (Table 5). There was no difference in the actual and predicted preoperative EQ-5D for the validation cohort (Table 4). There was, however, a significantly (*p* < 0.001) greater predicted postoperative (MD = 0.010, 95% CI 0.007 to 0.015), change (MD = 0.035, 0.029 to 0.042) and combined EQ-5D (MD = 0.013, 95% CI 0.010 to 0.017) relative to the actual scores for the validation cohort, but these differences were all less than the MCID (Table 3).

Predicting an individual’s EQ-5D utility from their OHS was less reliable. Prediction of an individual’s EQ-5D within +/−0.101 points ranged from 27.7% (the change in EQ-5D model) to 65.0% (the postoperative model; Table 6). The validation cohort had a significantly greater likelihood of predicting the EQ-5D to within +/−0.101 when compared to the modelling cohort (Table 6).

## 4. Discussion

This study demonstrated that the mean EQ-5D-3L utility index can be reliably predicted from the OHS at the cohort level. The preoperative, postoperative, change, and combined scores exhibited different distributions, and separate prediction models were developed for each. Statistically significant differences were observed between actual and predicted utilities for the postoperative, change, and combined models. However, these differences were below the MCID and were therefore not considered clinically meaningful. Incorporating individual OHS item responses resulted in marginal improvements in model performance. Some items showed little association with EQ-5D utility, while others contributed more strongly to prediction. At the individual patient level, however, predictive accuracy within ±0.101 (the MCID) varied substantially, ranging from 27.7% for the change model to 65.0% for the postoperative model. These findings provide a robust method to calculate quality of life scores from a joint specific score, allowing the efficacy of evolving technologies to be more widely evaluated. However, we would still recommend the gold standard of direct EQ-5D collection where feasible.

Several limitations of this study should be acknowledged. The reporting of mean and standard deviation was employed throughout to allow comparisons with the existing literature and minimal detectable change thresholds, despite non-normal pre- and postoperative data distributions. Although a statistically significant correlation was observed between the OHS and EQ-5D utility, a strong correlation (r > 0.7) was evident only for postoperative scores. This is not unexpected, as the OHS and EQ-5D measure different constructs of health and function; consequently, strong correlations may be less likely for preoperative scores and change scores [17]. Nevertheless, correlation coefficients exceeded 0.6 for these measures, indicating a moderate-to-strong association. Previous mapping studies have modelled individual OHS items against the EQ-5D utility. Although this approach was also explored in the current study, it resulted in only marginal improvements in model performance. Consequently, a more pragmatic approach was adopted, generating a single coefficient for each dataset assessed, which was considered more practical and easier to implement. Similarly, only one modelling approach was employed, a generalised linear model, allowing a single, consistent set of coefficients to be derived. This model has previously been shown to perform optimally in mapping studies of this type [9]. While these methodological choices may be viewed as limitations, they did not appear to compromise predictive accuracy when compared with studies that used more complex models incorporating multiple item-level coefficients and alternative modelling approaches. Additional patient-related factors, such as age, gender and BMI, may influence mapping performance [18]; however, inclusion of these variables did not improve the model and would have resulted in more complex algorithms, which may warrant further investigation.

The regression models defined by this study used similar techniques to the transfer to utility (TTU) models presented by Pinedo-Villanueva et al., predicting EQ-5D index both from the overall OHS score and from individual item responses [8]. However, important distinctions between our models and those described by Pinedo-Villanueva et al. arise from the handling of pre- and postoperative data. All data in their study were combined, despite the differing distributions observed in both the OHS and EQ-5D index scores collected at these distinct timepoints. Whilst the combined model described here was able to accurately predict EQ-5D index using combined pre- and postoperative data, this resulted in different model coefficients and poorer predictive accuracy at both the group and individual level than when these cohorts were considered separately. Contrastingly, their response mapping technique predicted EQ-5D health profiles rather than index scores, using a combination of multinomial logistic regression and simulation techniques, allowing subsequent conversion to the EQ-5D index using the corresponding population-specific tariff [8]. However, this approach generated a complex model, and the risk of inaccuracy in individual item predictions within even one EQ-5D domain can have significant impacts on the final index score [19].

We found that the mapping models reported in this study were able to accurately predict cohort-level EQ-5D from OHS data but were less accurate in predicting individual patient scores. The limited accuracy in individual EQ-5D index prediction was most obvious in the preoperative and change scores, with the postoperative model still able to reliably predict 65% of individual scores to within the MCID. This is similar to findings from studies previously mapping the EQ-5D index from other lower-limb joint-specific function scores, including the Western Ontario and McMaster Universities Osteoarthritis Index (WOMAC) and the Oxford Knee Score (OKS), where EQ-5D prediction for individual patient scores was more variable than that for the whole cohort [20,21]. However, given the established test–retest variation observed in EQ-5D scores for patients with hip osteoarthritis, it is possible that this individual prediction error may represent variability in the EQ-5D as a measurement instrument in this population [22]. It has also been proposed that mapping EQ-5D utilities from the OHS performs less well for cases with utility scores at the lower extreme of the scale [17]. This may partially explain the relatively limited accuracy observed in individual preoperative EQ-5D prediction, given the significant quality-of-life burden observed preoperatively whilst awaiting THA [14].

Cohort-level EQ-5D prediction using the preoperative, postoperative, change and combined models was highly accurate, with mean differences ranging from 0.01 (preoperative) to 0.035 (change). Whilst predicted postoperative, change and combined models were significantly different from observed EQ-5D, this difference was considerably below the MCID (0.101) and so considered not clinically relevant. Overall differences between predicted and actual index scores of 0.0005 to 0.0042 were reported by Pinedo-Villanueva et al., although the study validation cohort potentially underrepresented postoperative scores (less than 10% of the sample), and postoperative scores were typically overestimated by up to 0.03 [8]. Previous studies have reported mean absolute errors (MAEs) of 0.1 to 0.13 for models predicting EQ-5D from OHS in a Spanish population, typically exceeding the EQ-5D MCID [9]. Clinically meaningful values for the EQ-5D have been reported in a range of populations but are known to be dependent on both the population assessed and the procedure undergone [23,24]. The EQ-5D MCID following THA has been reported to be 0.101 using anchor-based methods in a UK NHS population, which was used as a threshold to assess the clinical relevance of score changes between groups in this study [15].

Whilst the EQ-5D mapping described in the current study allows the accurate estimation of cohort-level data from the OHS, it is not ideal for individual-level EQ-5D data. However, there are scenarios where EQ-5D data are not available, and collection may not be possible, such as when analysing historical data. The OHS has been widely collected in both clinical trial and registry settings since its creation in 1996 [1,25,26]. Where OHS data alone are available, the mapping of EQ-5D data could not only allow the estimation of historical quality-of-life metrics but also enable the extension of cost-utility analyses into the longer term for patients following THA [8]. The calculation of QALYs from historical patient cohorts where EQ-5D data were not collected at baseline may allow the more widespread evaluation of novel technologies, such as robotic-assisted THA. The conversion algorithms reported in this study are deliberately parsimonious, using only a coefficient and intercept for all final models, enabling the straightforward prediction of the EQ-5D from OHS scores. This provides practical benefits to clinicians and researchers seeking to calculate quality-of-life metrics in the absence of more complete data, but direct EQ-5D collection from patients remains the gold standard [27].

Disaggregating the OHS into individual item responses was shown to yield modest but consistent gains in the accuracy of EQ-5D utility estimation across the perioperative pathway. Notably, pain severity and the ability to shop emerged as the most influential predictors of HRQoL, which is a novel finding. Conversely, items relating to limping, night pain, and putting on socks showed limited contribution preoperatively or to change scores, suggesting potential ceiling effects or reduced relevance to generic health utility in these contexts.

## 5. Conclusions

The EQ-5D-3L utility index on a cohort level may be reliably estimated from the OHS. Predicted individual patient utility error beyond the MCID varied from one-third to more than two-thirds depending on the data assessed and so should not be employed for this purpose on a patient level.

## Figures and Tables

**Table 1:** Generalised linear regression analysis for the association of the preoperative, postoperative and change in Oxford hip score and EQ-5D index.

Timepoint	n	Variable	Beta (95% CI)	*p*-Value
**Preoperative**	3852	Constant	−0.142 (−0.162 to −0.121)	<0.0001
(R^2^ = 0.454)		OHS	0.026 (0.025 to 0.026)	<0.0001
RMSE = 0.25; MAE = 0.20				
**Postoperative**	3981	Constant	−0.075 (0.114 to 0.143)	<0.0001
(R^2^ = 0.618)		OHS	0.022 (0.021 to 0.022)	<0.0001
RMSE = 0.16; MAE = 0.11				
**Change**	3837	Constant	−0.007 (−0.025 to 0.012)	0.483
(R^2^ = 0.385)		OHS	0.022 (0.021 to 0.022)	<0.0001
RMSE = 0.28; MAE = 0.23				
**Combined**	7877	Constant	−0.087 (−0.099 to −0.075)	<0.001
(R^2^ = 0.659)		OHS	0.022 (0.022 to 0.023)	<0.001
RMSE = 0.13; MAE = 0.09				

**Table 2:** Generalised linear regression analysis for the association of the preoperative, postoperative and change in Oxford hip score according to the individual questions assessed and the associated EQ-5D index for these.

Oxford Hip Score Question	Preoperative (R^2^ 0.475)		Postoperative (R^2^ 0.628)		Change (R^2^ 0.400)	
	Beta (95%CI)	*p*-Value	Beta (95%CI)	*p*-Value	Beta (95%CI)	*p*-Value
**Q1:** Pain	0.091 (0.077 to 0.104)	<0.001	0.041 (0.033 to 0.048)	0.000	0.049 (0.038 to 0.059)	0.000
**Q2:** Washing	0.032 (0.021 to 0.043)	0.000	0.026 (0.017 to 0.036)	0.000	0.028 (0.016 to 0.039)	0.000
**Q3:** Transport	0.014 (0.000 to 0.028)	0.048	0.007 (−0.003 to 0.016)	0.168	0.013 (0.000 to 0.026)	0.044
**Q4:** Socks	0.008 (−0.002 to 0.018)	0.121	0.010 (0.004 to 0.017)	0.002	0.001 (−0.009 to 0.011)	0.792
**Q5:** Shopping	0.038 (0.029 to 0.047)	0.000	0.043 (0.037 to 0.049)	0.000	0.041 (0.032 to 0.051)	0.000
**Q6:** Walking	0.021 (0.012 to 0.030)	0.000	0.009 (0.003 to 0.015)	0.004	0.013 (0.005 to 0.021)	0.001
**Q7:** Stairs	0.019 (0.007 to 0.032)	0.002	0.031 (0.023 to 0.040)	0.000	0.018 (0.006 to 0.031)	0.003
**Q8:** Standing	0.019 (0.007 to 0.032)	0.002	0.023 (0.013 to 0.032)	0.000	0.031 (0.019 to 0.042)	0.000
**Q9:** Limping	0.003 (−0.006 to 0.012)	0.467	0.012 (0.006 to 0.019)	0.000	0.008 (0.000 to 0.016)	0.059
**Q10:** Severe Pain	0.023 (0.015 to 0.031)	0.000	0.009 (0.002 to 0.017)	0.016	0.024 (0.016 to 0.032)	0.000
**Q11:** Working	0.041 (0.028 to 0.053)	0.000	0.022 (0.012 to 0.032)	0.000	0.029 (0.017 to 0.041)	0.000
**Q12:** Night pain	0.004 (−0.004 to 0.013)	0.318	0.020 (0.013 to 0.027)	0.000	0.005 (−0.003 to 0.014)	0.210

**Table 3:** Associated EQ-5D-3L utility according to preoperative (pre), postoperative (post), change, and combined models using the OHS.

OHS	Associated EQ-5D-3L Utility				OHS	Associated EQ-5D-3L Utility			
	Pre	Post	Change	Combined		Pre	Post	Change	Combined
1	−0.116	−0.053	0.022	−0.065	25	0.508	0.475	0.550	0.463
2	−0.09	−0.031	0.044	−0.043	26	0.534	0.497	0.572	0.485
3	−0.064	−0.009	0.066	−0.021	27	0.56	0.519	0.594	0.507
4	−0.038	0.013	0.088	0.001	28	0.586	0.541	0.616	0.529
5	−0.012	0.035	0.110	0.023	29	0.612	0.563	0.638	0.551
6	0.014	0.057	0.132	0.045	30	0.638	0.585	0.660	0.573
7	0.040	0.079	0.154	0.067	31	0.664	0.607	0.682	0.595
8	0.066	0.101	0.176	0.089	32	0.690	0.629	0.704	0.617
9	0.092	0.123	0.198	0.111	33	0.716	0.651	0.726	0.639
10	0.118	0.145	0.220	0.133	34	0.742	0.673	0.748	0.661
11	0.144	0.167	0.242	0.155	35	0.768	0.695	0.770	0.683
12	0.170	0.189	0.264	0.177	36	0.794	0.717	0.792	0.705
13	0.196	0.211	0.286	0.199	37	0.820	0.739	0.814	0.727
14	0.222	0.233	0.308	0.221	38	0.846	0.761	0.836	0.749
15	0.248	0.255	0.330	0.243	39	0.872	0.783	0.858	0.771
16	0.274	0.277	0.352	0.265	40	0.898	0.805	0.880	0.793
17	0.300	0.299	0.374	0.287	41	0.924	0.827	0.902	0.815
18	0.326	0.321	0.396	0.309	42	0.950	0.849	0.924	0.837
19	0.352	0.343	0.418	0.331	43	0.976	0.871	0.946	0.859
20	0.378	0.365	0.440	0.353	44	1.002	0.893	0.968	0.881
21	0.404	0.387	0.462	0.375	45	1.028	0.915	0.990	0.903
22	0.430	0.409	0.484	0.397	46	1.054	0.937	1.012	0.925
23	0.456	0.431	0.506	0.419	47	1.080	0.959	1.034	0.947
24	0.482	0.453	0.528	0.441	48	1.106	0.981	1.056	0.969

OHS, Oxford Hip Score; EQ-5D-3L, Euroqol 5-Dimension 3-Level utility.

**Table 4:** The difference in the actual and predicted (from the OHS) preoperative, postoperative, change, and combined EQ-5D utilities for both the modelled data and the validation data cohorts.

Prediction Group	Actual	Predicted	Mean Difference (95% CI)	*p*-Value
**Modelling Cohort**				
**Preoperative**	0.390 (0.341)	0.400 (0.233)	0.009 (0.002 to 0.017)	0.022
(n = 3852)				
**Postoperative**	0.788 (0.253)	0.795 (0.201)	0.007 (0.002 to 0.011)	0.004
(n = 3981)				
**Change**	0.397 (0.360)	0.413 (0.204)	0.015 (0.006 to 0.024)	0.001
(n = 3837)				
**Combined**	0.592 (0.359)	0.581 (0.287)	0.012 (0.007 to 0.017)	<0.001
(n = 7833)				
**Validation Cohort**				
**Preoperative**	0.367 (0.335)	0.367 (0.233)	0.001 (−0.005 to 0.006)	0.828
(n = 7168)				
**Postoperative**	0.820 (0.237)	0.831 (0.180)	0.010 (0.007 to 0.015)	<0.001
(n = 6101)				
**Change**	0.423 (0.335)	0.458 (0.221)	0.035 (0.029 to 0.042)	<0.001
(n = 5629)				
**Combined**	0.576 (0.370)	0.562 (0.303)	0.013 (0.010 to 0.017)	<0.001
(n = 13,269)				

**Table 5:** Comparison of baseline demographics and patient-reported outcome measures (PROMs) between the two cohorts.

Demographic		Cohort	
		Modelling	Validation
**Gender** (n, % of group)	Male	1622 (40.6)	2798 (39.0)
	Female	2375 (59.4)	4370 (61.0)
**Age** (years: mean, SD)		68.3 (11.6)	67.8 (11.9)
**PROMs** (mean, SD)			
**Preoperative**	**OHS**	20.8 (9.0)	19.6 (9.0)
	**EQ5D**	0.391 (0.341)	0.366 (0.336)
**Postoperative**	**OHS**	39.6 (9.1)	41.1 (8.2)
	**EQ5D**	0.788 (0.253)	0.818 (0.238)
**Change**	**OHS**	18.8 (10.4)	20.8 (10.0)
	**EQ5D**	0.397 (0.362)	0.421 (0.335)
**Combined**	**OHS**	30.3 (13.0)	29.5 (13.8)
	**EQ5D**	0.691 (0.360)	0.691 (0.371)

OHS, Oxford Hip Score; EQ-5D, Euroqol 5-Dimension 3-Level utility; PROMs, patient-reported outcome measures; SD, standard deviation.

**Table 6:** Predicted EQ-5D-3L utility within 0.101 of the actual utility according to timepoint assessed, combined (pre- and postoperative) scores and change in score.

Timepoint	Modelling Cohort		Validation Cohort		OR (95% CI)	*p*-Value
	Yes (n, %)	No (n, %)	Yes (n, %)	No (n, %)		
**Preoperative**	1076 (27.9)	2776 (72.1)	2171 (30.3)	4997 (69.7)	1.12 (1.03 to 1.22)	0.010
**Postoperative**	2373 (59.6)	1608 (40.4)	3968 (65.0)	2133 (35.0)	1.26 (1.16 to 1.37)	<0.001
**Change**	969 (25.3)	2867 (74.7)	1558 (27.7)	4063 (72.3)	1.13 (1.03 to 1.25)	0.008
**Combined**	3330 (42.5)	4506 (57.5)	5833 (44.0)	7436 (56.0)	1.06 (1.00 to 1.12)	0.038

OR, odds ratio; 95% CI, 95% confidence interval.

## Data Availability

Materials used in this study are available from the authors on reasonable request.

## Supplementary Materials

The following supporting information can be downloaded at: https://www.mdpi.com/article/10.3390/jcm15218268/s1, Table S1: Mapping onto Preference-based measures reporting Standards (MAPS) Checklist.
