# Supplementary material for: Mapping Analysis to Predict the Associated EQ-5D-3L Utility Values from the Oxford Hip Score
Source: J Clin Med. 2026 Oct 23;15(21):8268. doi: 10.3390/jcm15218268 (PMC13763906; doi:10.3390/jcm15218268)
Supplement: Supplementary file 1 [file jcm-15-08268-s001.zip › jcm-4564829-supplementary.pdf]

Section/topic	Item number	Recommendation:	Reported on:
Title and abstract			
Title	1	Identify the report as a study mapping between outcome measures. State the source measure(s) and generic, preference-based target measure(s) used in the study.	Title
Abstract	2	Provide a structured abstract including, as applicable: objectives; methods, including data sources and their key characteristics, outcome measures used and estimation and validation strategies; results, including indicators of model performance; conclusions; and implications of key findings.	Abstract
Introduction			
Study rationale	3	Describe the rationale for the mapping study in the context of the broader evidence base.	Pg 3. Para 2.
Study objective	4	Specify the research question with reference to the source and target measures used and the disease or population context of the study.	Pg 4. Para 1.
Methods			
Estimation sample	5	Describe how the estimation sample was identified, why it was selected, the methods of recruitment and data collection, and its location(s) or setting(s).	Pg 4. Para 3.
External validation sample	6	If an external validation sample was used, the rationale for selection, the methods of recruitment and data collection, and its location(s) or setting(s) should be described.	Pg 4. Para 3.
Source and target measures	7	Describe the source and target measures and the methods by which they were applied in the mapping study.	Pg 5. Para 1.
Exploratory data analysis	8	Describe the methods used to assess the degree of conceptual overlap between the source and target measures.	Pg 5. Para 2.

Section/topic	Item number	Recommendation:	Reported on:
Missing data	9	State how much data were missing and how missing data were managed in the sample(s) used for the analyses.	Pg 4, Para 3.<br>Pg 5, Para 2.
Modelling approaches	10	Describe and justify the statistical model(s) used to develop the mapping algorithm.	Pg 5, Para 3.
Estimation of predicted scores or utilities	11	Describe how predicted scores or utilities are estimated for each model specification.	Pg 5, Para 3.<br>Pg 6, Para 1.
Validation methods	12	Describe and justify the methods used to validate the mapping algorithm.	Pg 6, Para 1.
Measures of model performance	13	State and justify the measure(s) of model performance that determine the choice of the preferred model(s) and describe how these measures were estimated and applied.	Pg 5, Para 3.<br>Pg 6, Para 1.
Results			
Final sample size(s)	14	State the size of the estimation sample and any validation sample(s) used in the analyses (including both number of individuals and number of observations).	Pg 4, Para 3.
Descriptive information	15	Describe the characteristics of individuals in the sample(s) (or refer back to previous publications giving such information). Provide summary scores for source and target measures, and summarize results of analyses used to assess overlap between the source and target measures.	Table 5.
Model selection	16	State which model(s) is(are) preferred and justify why this(these) model(s) was(were) chosen.	Pg 7, Para 4.
Model coefficients	17	Provide all model coefficients and standard errors for the selected model(s). Provide clear guidance on how a user can calculate utility scores based on the outputs of the selected model(s).	Pg 7, Para 4.<br>Table 3.

Section/topic	Item number	Recommendation:	Reported on:
Uncertainty	18	Report information that enables users to estimate standard errors around mean utility predictions and individual-level variability.	Table 1.
Model performance and face validity	19	Present results of model performance, such as measures of prediction accuracy and fit statistics for the selected model(s) in a table or in the text. Provide an assessment of face validity of the selected model(s).	Table 4.
<u>Discussion</u>			
Comparisons with previous studies	20	Report details of previously published studies developing mapping algorithms between the same source and target measures and describe differences between the algorithms, in terms of model performance, predictions and coefficients, if applicable.	Pg 3, Para 2<br>Pg 10 Para 2-3
Study limitations	21	Outline the potential limitations of the mapping algorithm.	Pg 9, Para 2
Scope of applications	22	Outline the clinical and research settings in which the mapping algorithm could be used.	Pg 12, Para 1
Other			
Additional information	23	Describe the source(s) of funding and non-monetary support for the study, and the role of the funder(s) in its design, conduct and report. Report any conflicts of interest surrounding the roles of authors and funders.	None
